# Mini-Basketball Training Program Improves Social Communication and White Matter Integrity in Children with Autism

**DOI:** 10.3390/brainsci10110803

**Published:** 2020-10-31

**Authors:** Kelong Cai, Qian Yu, Fabian Herold, Zhimei Liu, Jingui Wang, Lina Zhu, Xuan Xiong, Aiguo Chen, Patrick Müller, Arthur F. Kramer, Notger G. Müller, Liye Zou

**Affiliations:** 1 College of Physical Education, Yangzhou University, Yangzhou 225127, China; MX120170353@yzu.edu.cn (K.C.); MX120170354@yzu.edu.cn (Z.L.); MX120170362@yzu.edu.cn (J.W.); DX120190066@yzu.edu.cn (X.X.); 2 Exercise and Mental Health Laboratory, Institute of Mental Health, School of Psychology, Shenzhen University, Shenzhen 518060, China; yuqianmiss@163.com (Q.Y.); liyezou123@szu.edu.cn (L.Z.); 3 Research Group Neuroprotection, German Center for Neurodegenerative Diseases (DZNE), Leipziger Str. 44, 39120 Magdeburg, Germany; Fabian.herold@dzne.de (F.H.); patrick.mueller@dzne.de (P.M.); notger.mueller@dzne.de (N.G.M.); 4 School of Physical Education and Sports Science, Beijing Normal University, Beijing 100875, China; zhulina827@mail.bnu.edu.cn; 5 Center for Cognitive and Brain Health, Department of Psychology, Northeastern University, Boston, MA 02115, USA; a.kramer@northeastern.edu; 6 Beckman Institute, University of Illinois at Urbana-Champaign, Champaign, IL 61801, USA

**Keywords:** exercise intervention, autism spectrum disorders, social communication, white matter integrity, diffusion tensor imaging

## Abstract

Impairments in social communication (SC) represent one of the core symptoms of autism spectrum disorder (ASD). While previous studies have demonstrated that exercise intervention improves SC in children with ASD, there is currently no neuroscientific evidence supporting its benefits. Therefore, we evaluated the outcomes of a long-term exercise intervention on SC and white matter integrity (WMI) in children with ASD, and further explored the neural mechanism of exercise intervention on SC in these children. Twenty-nine children aged 3–6 years with ASD were assigned to either exercise group (*n* = 15) or control group (*n* = 14). The exercise group received a scheduled mini-basketball training program (5 sessions per week, forty minutes per session) for 12 consecutive weeks, while the control group was instructed to maintain their daily activities. Groups were assessed before and after intervention on SC and WMI. SC scores were lower in the exercise group post-intervention. Compared with the control group, WMI of the exercise group showed higher fractional anisotropy in the body of corpus callosum, fornix, right cerebral peduncle, left posterior limb of internal capsule, right retrolenticular part of internal capsule, left anterior corona radiate and left superior fronto-occipital fasciculus; lower mean diffusivity in the left anterior corona radiate and the bilateral corticospinal tract. Furthermore, increased WMI was associated with lower scores on a measure of social cognition in the overall sample. This study is the first to provide evidence that exercise intervention improves SC and white matter integrity in children with autism.

## 1. Introduction

Autism spectrum disorders (ASD) are a group of neurodevelopmental disabilities with specific cognitive-behavioral features and fairly prevalent comorbidities (i.e., epilepsy, depression, and attention-deficit/hyperactivity disorder) [1]. The word “autism” originated from the Greek word “auto” which means “self” in English. Accordingly, ASD children are typically self-absorbed and live in a seemingly private world with limited social communication (SC) [2]. Such SC deficits involve social cognition, pragmatics, language processing, and verbal and non-verbal communication [3]. Furthermore, “persistent deficits in SC and interaction” as one core symptom of ASD are used as the main diagnostic criterion of ASD in both the International Classification of Diseases—11th Revision [4] and the Diagnostic and Statistical Manual—Fifth Edition (DSM-5) [5]. Notably, SC deficits among these children resulted in poor attachment and intimacy, delayed learning process, and reduced self-esteem and self-confidence, which, in turn, has negative impacts on all aspects of their life and their future career [6]. In this context, epidemiological data show an increasing prevalence of ASD of 1.85% in children in 2016 worldwide, particularly among boys [7]. This increased prevalence of ASD and the associated disease burden have motivated several scholars to extend the research regarding autistic etiology, diagnosis, and treatment [1].

Up to now, various evidence-based ASD interventions (applied behavior analysis [8], discrete trial training [9], and pivotal response training [10]) that aimed to improve SC have been applied successfully, but to maximize such positive treatment effects, researchers have also started to encourage the use of physical exercise training programs as an adjuvant therapy for ASD children within usual care treatments [11,12,13]. Specifically, researchers found positive effects on antisocial behaviors [12] and SC-related outcomes (e.g., emotion regulation, social awareness, social cognition, social communication, and mannerisms) among ASD patients aged 3 to 12 years [11] following different physical training programs (e.g., swimming, jogging, martial arts, and basketball [14,15,16,17,18,19]. Among these physical training interventions, basketball training is deemed to be a promising intervention because this team sport requires individuals to set goals, make decisions, communicate, and manage conflicts in a supportive and trusting atmosphere which, in turn, might help to alleviate the SC deficits of children suffering from ASD. Indeed, it was observed that a specific basketball training program has superior effects on SC deficits as compared to the purely physical exercises (e.g., jogging) [11,12,13]. Thus, physically and communicatively demanding team sport exercises such as basketball seem to be a promising intervention strategy to lower SC deficits in ASD children. Consequently, this line of research seems a promising field for further investigation.

In this context, it is also important to understand the potential neurobiological correlates of ASD and the possible effect of physical training interventions on them. The available evidence strongly suggests that ASD is associated with structural brain changes, especially in white matter integrity (WMI) [20], that can be sensitively assessed by diffusion tensor imaging (DTI). Indeed, this neuroimaging method has been widely applied in the research in autism spectrum disorders [21,22,23] and extensive neuropsychological and neuroimaging evidence indicated that abnormalities of WMI in ASD children were associated with SC impairments [21,22,23]. Specifically, the lower fractional anisotropy (FA; assessing the amount of water diffusion anisotropy within tissues) and higher mean diffusion (MD; assessing water diffusion within tissues) values of DTI in the prefrontal lobe, temporal lobe, and corpus callosum are associated with deficits in complex socio-emotional and communication domains in ASD children [23]. Collectively, these findings suggest that white matter abnormalities play a key role in the development of SC deficits in ASD children. Interestingly, several studies have documented that children with a higher physical fitness level (e.g., cardiorespiratory fitness) and children who regularly engage in physical exercise interventions exhibit superior white matter integrity, which strongly supports the idea of integrating physical training interventions as an adjuvant intervention in the therapy of children suffering from ASD. However, this field of research is far less explored and more empirical evidence is needed to buttress these assumptions. Hence, this study aims (i) to investigate whether a mini-basketball training program (MBTP; basketball training designed for children), serving as adjuvant therapy, could improve SC deficits; (ii) to evaluate whether MBTP can influence WMI in ASD children by analyzing WMI changes via DTI; and (iii) to examine possible neurobehavioral relationships between physical exercise-induced WMI changes and SC deficits.

## 2. Methods

### 2.1. Study Design

The study with quasi-experimental design was conducted between October and December 2018 in Yangzhou, China. Approval letters have been received from both the Ethics Committee of Yangzhou Maternal and Child Health Hospital (No. 201806001) and the Ethics and Human Protection Committee of the Affiliated Hospital of Yangzhou University. Meanwhile, this study was registered with the Chinese Clinical Trial Registry (ChiCTR1900024973) on 5 August 2019, prior to the beginning of experiments. Ethics approval from the Ethics and Human Protection Committee of the Affiliated Hospital of Yangzhou University was fully disclosed to all participants and their guardians, and written informed consent was obtained from each participant’s guardian according to the provision of the Declaration of Helsinki.

### 2.2. Participants

Ninety-four children (aged 3–6 years) diagnosed with ASD via DSM-5 were recruited from Chuying Child Development Center and Starssailor Education Institution (Yangzhou, China), which established good cooperative relations with the Affiliated Hospital of Yangzhou University. All participants were students of these two institutions and their caregivers had the desire to let their children to receive free DTI assessment that was beneficial to the further treatment. The exclusion criteria for participants were: (1) involvement in structured exercise program in the past 6 months; (2) co-morbid psychiatric (i.e., attention deficit and hyperactivity disorder) and/or neurological disorders (i.e., epilepsy, phenylketonuria, fragile X syndrome, tuberous sclerosis); (3) visual and/or auditory impairments; (4) history of head trauma; (5) medical contraindications to exercise (i.e., acute phase after operation or fracture). According to the above criteria and attendance, 59 eligible participants were included and assigned to either experimental group (*n* = 30; from the Starssailor Education Institution) or control group (*n* = 29; from Chuying Child Development Center) in terms of location. Of note, ASD children from the two different places and their parents had no opportunity to socially interact with one another in order to minimize differential expectancies. Because some ASD children did not complete the post-intervention DTI assessments, they were not considered, finally leading to a total of 29 participants for data analysis: experimental group (*n* = 15) and control group (*n* = 14).

### 2.3. Usual Care and Mini-Basketball Training Program

Both groups followed a standard rehabilitation program (Applied Behavior Analysis, ABA) as usual care [8]. No significant group difference was observed in rehabilitation duration for the experimental group (*Mean* = 4.47, *SD* = 0.99) and control group (*M* = 4.49, *SD* = 0.893). Besides, ASD children in the experimental group received an additional mini-basketball session, while the control group maintained an unchanged lifestyle.

The mini-basketball training program (MBTP) conducted by two certified physical educators was adopted from previous studies [24,25,26,27] and operational details can be found in our published articles [13,28]. A program with various levels of difficulty was designed (Table 1), and a collective teaching mode was used to facilitate social interaction and communication among participants while parent’s participation in and attendance at exercise sessions was strongly encouraged. Twelve-week MBTP was arranged for ASD children in the experimental group (40 min × 5 sessions per week × 12 weeks) and each session included four stages of (a) 5-min warm-up, (b) 20-min basic basketball skill learning, (c) 10-min basketball games, and (d) 5-min cool-down (Table 2). In the initial 2 weeks of MBTP, some simple mini-basketball games were arranged to develop children’s interest in basketball training and induce therapeutic effects of desensitization. After the 2-week program, most autistic children were motivated toward the participation of further mini-basketball training. Even though some autistic individuals were less motivated, they could still complete the entire training process well. The average heart rate was monitored during the MBTP intervention (MD = 135.97, SD = 6.053).

### 2.4. Behavioral Measurements

At baseline, demographic information (age, gender, height, weight, and body mass index (BMI)) were obtained. Childhood Autism Rating Scale (CARS) [29] and clinical assessment report were used to confirm the ASD severity. The CARS includes 15 items (14 items for assessing autistic behaviors and 1 item for assessing the general impression of ASD), with each item scoring from one to four (higher score indicating more severe impairment). A score above 30 is regarded as a sign of autism (mild-to-moderate autism: 30–36.5; severe autism: 37–60) [29].

Due to the influences of sleep on ASD syndrome [30,31], sleep problems were assessed by the Children’s Sleep Habits Questionnaire (CSHQ) [32]. The CSHQ is a parent-rated questionnaire which assesses the frequency of eight behaviors (bedtime resistance, sleep onset delay, sleep duration, sleep anxiety, night waking, parasomnias, sleep disordered breathing, and daytime sleepiness) related to children’s sleep difficulties. A total score over 41 indicates the sleep disorder [30,31].

Regarding the influences of diet on ASD syndrome, eating behaviors were assessed by the parent-reported Child Eating Behavior Questionnaire (CEBQ) [33]. The CEBQ consists of eight subscales (food responsiveness, emotional over-eating, enjoyment of food, desire to drink, satiety responsiveness, slowness in eating, emotional under-eating, and food fussiness) covering 35 items. The responses were scored from 1 to 5 and the average of each subscale was calculated if at least 80% of the items were completed.

Social Responsiveness Scale—Second Edition (SRS-2) [34], a 65-item and 4-point frequency scale, was used to measure the changes in the severity of SC impairments between pre- and post-intervention. There are five subscales in total (social awareness, social cognition, social communication, social motivation, restrictive interests and repetitive behavior) and a higher total T-score means more severe impairment in SC (mild impairment: 60–65; moderate impairment: 66–75; severe impairment: above 76).

### 2.5. Diffusion Tensor Imaging Acquisition and Analysis

Images were acquired with a 3.0T GE Healthcare whole body high-speed imaging device equipped for echo planar imaging (GE Discovery MR750w 3.0T, Chicago, USA) in the Affiliated Hospital of Yangzhou University. Participants were required to lie in the supine position and received sedation for scanning. Head stabilization was achieved with foam pads and noises were attenuated via 29 dB-rating earplugs (OHROPAX, Germany). The DTI protocol was as followed: repetition time (TR) = 16,500 ms, echo time (TE) = 96.2 ms, flip angle = 90°, field of view (FOV) = 224 × 224 mm^2^, acquisition matrix size = 112 × 112, 70 interleaved slices, voxel size = 2 × 2 × 2 mm^3^, 3 B0 (B value = 0; B value is a value reflecting gradient effects) images, 30 diffusion weighted images, and b value = 1000 s/mm^2^.

Pipeline for Analyzing Brain Diffusion Images (PANDA) [35], a toolbox in MatLab, was used for fully automated processing of diffusion images. Main procedures included preprocessing and producing diffusion metrics in preparation for statistical analysis (local diffusion homogeneity = 7 voxels, normalizing resolution in smooth = 2 mm, and smoothing kernel = 6 mm). The preprocessing steps were executed serially, including converting DICOM (Digital Imaging and Communications in Medicine) files into Nifti images, estimating the brain mask, cropping raw images, correcting for the eddy-current effects, and calculating diffusion tensor metrics. Then, diffusion metrics (FA and MD) were normalized into the Montreal Neurological Institute (MNI) space via the atlas-based analysis, and regional diffusion metrics were calculated by averaging the values within each region of the ICBM (International Consortium of Brain Mapping) DTI-81 atlas.

### 2.6. Statistical Analyses

Statistical analyses were conducted using SPSS (Statistical Package for the Social Sciences) Version 20.0 (IBM, Armonk, NY, USA). Means and standard deviations were calculated for all variables and a significance parameter of *p* < 0.05 was adopted. Effect size was computed and reported as a partial *η*^2^ value for the ANOVAs.

Demographic variables were compared between the experimental and control groups, with independent sample *t*-tests for continuous variables and *χ*^2^ tests for sex proportion. To assess the effects of the MBPT on social communication performance, we conducted ANOVA analysis with a 2 (time: baseline vs. post-test) × 2 (group: control group vs. experimental group) repeated measures analysis. In the case of significant interaction effects, post-hoc tests were performed. To investigate whether MBTP has a global impact on brain WMI, repeated measures ANOVAs were conducted for both FA and MD to examine the effects of their interactions; post-hoc analyses were conducted with planned pairwise comparisons when significant interaction effects were revealed.

An exploratory analysis was conducted to investigate whether improved white matter integrity was associated with improved social communication in the overall sample. For each white matter variable that showed a significant time-by-group interaction, the change from baseline to post-test (*z*FA and *z*MD) was calculated for each individual. These difference scores were then entered into two separate *k*-means clustering analyses (*k* = 2, input: *z*FA and *z*MD; ≤10 iterations). Student’s *t*-test evaluated differences in SRS-2 performance between clusters. The analyzed dependent variables were the SRS-2 score and whole-brain white matter integrity (FA and MD).

## 3. Results

### 3.1. Demographic Analyses

There were no significant differences between the groups in demographic variables (including age, gender, height, weight, BMI, and CARS, CSHQ, and CEBQ; Table 3) and SRS-2 (Table 4) at baseline.

### 3.2. Social Communication Performance

For SC total score, between-group significance was not observed (*p* < 0.05). A group × time interaction effect was observed on the SC total score (F (1, 27) = 11.869 [F = F value in the ANOVA analysis], *p* < 0.01, partial *η*^2^ = 0.305). Follow-up results indicated that SC total scores of the experimental group at the post-test were significantly lower relative to baseline (F (1, 27) = 5.525, *p* < 0.05), whereas such a positive effect was not observed in the control group. Of note, reduction in the SC total score indicates better performance.

Group-by-time interaction effect was only observed in the three subscales (social cognition, social communication, and autistic mannerisms—reduced point indicates better performance: F (1, 27) = 11.872, 10.094, 6.283, *p* < 0.05, partial *η*^2^ = 0.305, 0.272, 0.189, respectively). Follow-up results for the subscales are presented below: (1) the post-test social cognition score was significantly lower than that at baseline in the experimental group (F (1, 27) = 8.591, *p* < 0.05), where no significant change from the post-intervention to pre-test was observed in the control group (F (1, 27) = 3.839, *p* > 0.05); (2) the post-test social communication score was significantly lower than that at baseline in the experimental group (F (1, 27) = 4.406, *p* < 0.05, reduction indicates better performance), whereas a higher point was observed in the control group from the post-test to pre-test (F (1, 27) = 5.712, *p* < 0.05; higher point indicates worse symptom); (3) there was no significant difference between baseline and post-test in the experimental group (F (1, 27) = 0.592, *p* > 0.05) in terms of autistic mannerisms, whereas the post-test score was significantly higher than that at baseline in the control group (F (1, 27) = 7.520, *p* < 0.05; greater score indicates worse symptoms).

### 3.3. White Matter Structure

Table 5 shows differences in FA and MD values for specific fiber tracts in an atlas-based ROI (region of interest) analysis between groups. There were no significant differences between groups in both FA and MD values at baseline.

**Figure 1:**
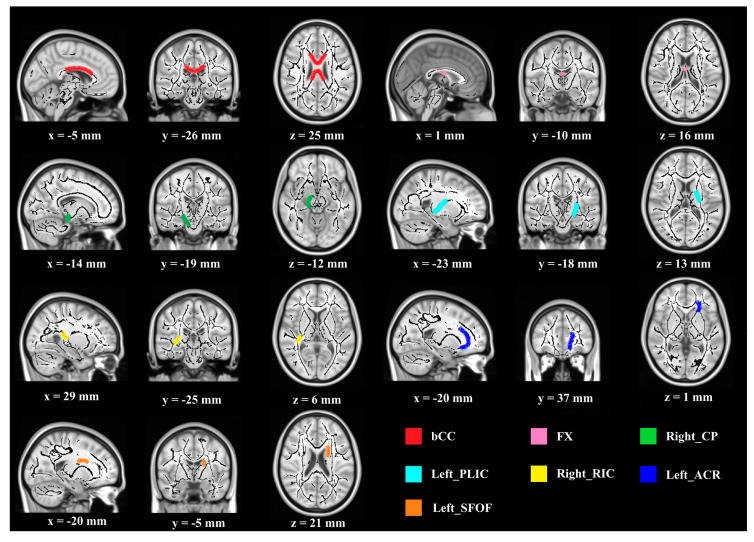
Demonstration of brain regions with significant FA increases after intervention. (Notes—Higher FA value indicates better SC function. Regions are overlaid on the FMRIB58_FA-skeleton and MNI152_T1 template. Abbreviations—FA: fractional anisotropy, bCC: body of corpus callosum, FX: fornix, CP: cerebral peduncle, PLIC: posterior limb of internal capsule, RIC: retrolenticular part of internal capsule, ACR: anterior corona radiate, SFOF: superior fronto-occipital fasciculus; “x, y, z” refer to “values of x-axis, y-axis, z-axis” in the x-y-z coordinates).

Regarding FA values, significant differences between groups were observed in the following seven WMI regions (Figure 1): body of corpus callosum (bCC) (F (1, 27) = 4.897, *p* < 0.05, partial *η*^2^ = 0.154), fornix (FX) (F (1, 27) = 7.020, *p* < 0.05, partial *η*^2^ = 0.206), right cerebral peduncle (CP) (F (1, 27) = 5.032, *p* < 0.05, partial *η*^2^ = 0.157), left posterior limb of internal capsule (PLIC) (F (1, 27) = 4.638, *p* < 0.05, partial *η*^2^ = 0.147), right retrolenticular part of internal capsule (RIC) (F (1, 27) = 6.911, *p* < 0.05, partial *η*^2^ = 0.204), left anterior corona radiate (ACR) (F (1, 27) = 4.543, *p* < 0.05, partial *η*^2^ = 0.144), and left superior fronto-occipital fasciculus (SFOF) (F (1, 27) = 5.950, *p* < 0.05, partial *η*^2^ = 0.181). Furthermore, compared with the FA value at the baseline, a higher FA value after the intervention was observed in the experimental group (higher FA indicates greater WMI improvement) (F (1, 27) = 5.615, 4.479, 6.921, 7.333, 9.652, 8.131, 5.399, *p* < 0.05). For the control group, no significant difference in FA value was shown pre- and post-intervention.

**Figure 2:**
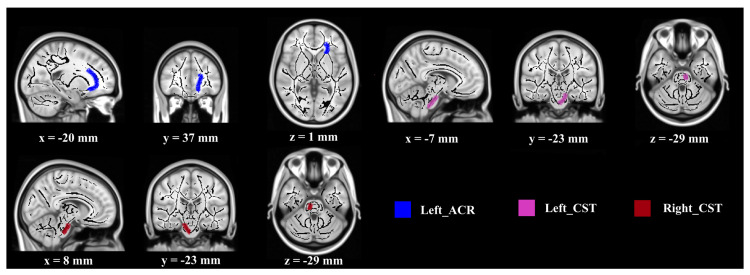
Demonstration of brain regions with significant MD increases after intervention. (Notes—Lower MD value indicates better SC function. Regions are overlaid on the FMRIB58_FA-skeleton and MNI152_T1 template. Abbreviations—MD: mean diffusion, ACR: anterior corona radiate, CST: corticospinal tract.).

Regarding MD values, significant differences between groups were found in the following three WM regions (Figure 2): right corticospinal tract (CST) (F (1, 27) = 5.956, *p* < 0.05, partial *η*^2^ = 0.181), left corticospinal tract (CST) (F (1, 27) = 5.024, *p* < 0.05, partial *η*^2^ = 0.157), and left anterior corona radiate (ACR) (F (1, 27) = 4.230, *p* < 0.05, partial *η*^2^ = 0.135). The post-intervention MD values were lower than that at baseline in the experimental group (F (1, 27) = 4.917, 3.398, 6.728, *p* < 0.05), but not in the control group.

### 3.4. Exploratory K-Means Analyses

**Figure 3:**
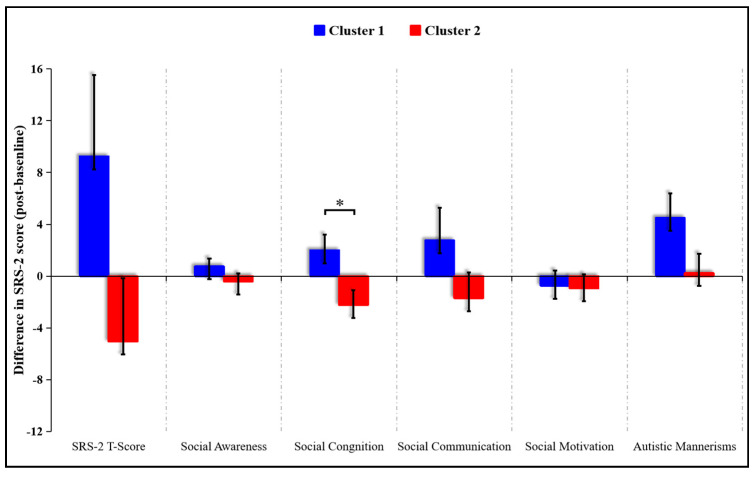
Illustrations of K-means cluster analyses (cluster 2 showed significantly lower scores on the social cognition subscale of the SRS-2 compared to cluster 1. * *p* < 0.05).

In order to examine the associations between WMI improvement and SC performance, exploratory K-means analyses were conducted. Participants were subdivided into two groups based on whether the WMI was improved (increased FA value and decreased MD value) or not. Two clusters (cluster 1 and cluster 2) were identified which significantly differed in the WMI pre- and post-intervention (cluster 1: three participants from experimental group and nine from control group; cluster 2: twelve participants from experimental group and five from control group). Cluster membership did not significantly overlap with exercise/control group membership (χ^2^ (1, *N* = 29) = 5.85, *p* = 0.016). Cluster 1 (not-improved WMI group) was composed of participants without WMI improvement (*n* = 12), and cluster 2 (improved WMI group) was composed of participants with WMI improvement (*n* = 17). Compared to cluster 1, a significantly lower score on the social cognition subscale was observed in cluster 2 (t (27) = 2.473, *p* = 0.02) (Figure 3). No significance was observed between the two clusters in SRS-2 total scores and other subscales.

## 4. Discussion

This study first assessed ASD children’s SC performance via two indices (FA and MD) of DTI, which well reflected pathological and developmental changes in axonal density and size, myelination, and organizational fiber coherence. Our findings demonstrated that MBTP has the potential to improve ASD children’s SC skills. Higher FA value (body of corpus callosum, fornix, right cerebral peduncle, left posterior limb of internal capsule, right retrolenticular part of internal capsule, left anterior corona radiate, and left superior fronto-occipital fasciculus) and lower MD value (left corticospinal tract, right corticospinal tract, and left anterior corona radiate) were associated with greater WMI improvement. Moreover, positive associations between changes in indices of white matter integrity and SC performance were found in the overall sample, which supports the effectiveness of MBTP by providing initial evidence for possible neural correlates eliciting, at least partly, the improvement of SC skills in children who are diagnosed to suffer from ASD.

Our findings related to corpus callosum and fornix are consistent with a substantial body of evidence from neuroimaging studies suggesting that the limbic structures are altered in ASD [36,37,38]. In the literature, it is also reported that abnormalities in limbic circuits correlated with social communication impairments being prevalent in individuals with ASD (i.e., antisocial behavior). This finding stresses the neurobiological basis of such impairments irrespective of the etiology of the disease [39,40,41]. As one of the essential components of the limbic system, the corpus callosum is the largest axonal pathway in the human brain and the main fiber tract transferring information [42] from one to the other brain hemisphere. Abnormalities in the corpus callosum were widely observed in neuropsychiatric disorders (e.g., ASD, bipolar disorder, schizophrenia) and are regarded as nonspecific manifestations of generalized deficits in interhemispheric connectivity [43,44]. Regarding ASD, some researchers proposed that early damage in the cingulate cortex led to callosal white matter reorganization and ultimately disordered interhemispheric communication which, in turn, contribute to the development of social communication deficits [45]. Notably, significant reductions in FA and MD of corpus callosum progressed with age and peaked at the mid-twenties among people diagnosed with ASD [46]. In our study, MBTP attenuated the progression of FA reduction (higher FA values indicating better SC performance) in limbic regions (corpus callosum and fornix) and enhances the social communication of ASD children. Speculatively, improved social communication skills in response to MBTP might have been caused by the increase in and preservation of the brain white matter microstructures in our cohort consisting of children with ASD. However, this assumption requires further well-designed studies using elaborated methods such as causal mediation analysis to provide support for such a relationship.

In addition, MBTP might improve social communication skills via its positive influence on the fronto-occipital fasciculus, which is, in general, negatively affected by ASD [47]. Considering the spatial conditions, the fronto-occipital fasciculus in ASD neurobiology is rather important for the processing of social information among ASD children [48]. The fronto-occipital fasciculus directly connected with the fusiform gyrus and associated with all components of the “social brain”: frontal (ventromedial prefrontal cortex), temporal (amygdala, superior temporal sulcus, temporoparietal junction), parietal (temporoparietal junction, somatosensory cortices), and occipital lobes [47]. Additionally, previous studies observed that lesions in the fronto-occipital fasciculus were associated with facial emotion recognition impairments (e.g., recognizing feelings such as fear, anger, and sadness), which is one of the key characteristics of ASD [49,50]. However, it is worth noting that, even though the fronto-occipital fasciculus is connected with all major modules in the social brain network, the change of a single fiber tract alone cannot explain the diversity of ASD clinical manifestations [47] and/or the improvements in behavioral symptoms in response to a specific treatment (e.g., MBTP).

Regarding subcortico-cortical tracts, increased FA values in internal capsule and middle cerebellar peduncle add to the limited body of DTI evidence on MBTP-induced benefits on social communication among ASD children. The internal capsule, the inferomedial part of the cerebral hemisphere, contained both ascending and descending axons between the basal ganglia and cerebral cortex. The anterior limb of the internal capsule, together with the genu, participated in the ASD dysfunctions in working memory directly and led to disconnection for auditory, visual, somatosensory, and attentional systems via thalamocortical fibers [51,52]. Robust impairments of the internal capsule, with significant reduction in FA, have been correlated with neuropsychological abnormalities (i.e., memory dysfunction) and motor impairments in ASD [53,54]. For example, Keller et al. [55] observed FA reduction in the right retrolenticular part of the internal capsule and Cheng et al. [20] detected reduced FA in the left posterior limb of the internal capsule. Accordingly, our findings suggested that MBTP can improve FA values in the two brain regions mentioned above. Additionally, increased FA value in the middle cerebellar peduncle was also noted in our sample in response to MBTP. The middle cerebellar peduncle appears to be responsible, in part, for coping and sending motor-execution commands to lower neurons through the pyramidal tract [56]. Structural and functional abnormalities (i.e., reduced FA) in the middle cerebellar peduncle have been correlated with motor and attentional impairments in various psychiatric disorders including ASD [57,58,59,60].

Moreover, the corticospinal tract, constituting a large part of the internal capsule, participates in the transfer of motor information between lower motor neurons and the primary motor cortex. The corticospinal tract above the basal ganglia was a part of the corona radiata. According to previous studies [61,62], motor abnormalities in ASD were often interpreted by impairments in the corona radiata and corticospinal tract, which were important motor pathways in the human brain [63]. Surprisingly, in our study, the improved MD in the corticospinal tract and corona radiata, as well as increased FA value, was associated with MBTP-induced social communication enhancement among ASD children. Definitely, this finding strengthens the important role of corticospinal tract and corona radiata on ASD abnormalities’ modulation.

Despite this study providing interesting findings and insights related to efficient treatment on SC among ASD children, some limitations have to be acknowledged. Firstly, the present study only focused on the improvement of FA and MD values, and other indicators of white matter integrity (i.e., radial and axial diffusivity) have not been explored. Thus, conclusions from these two indicators should be drawn cautiously. Secondly, MBTP was offered as an adjunctive treatment of existing interventions but not monotherapy, and the interaction among treatments and optimum therapeutic plan are relatively unexplored and not well-understood.

## 5. Conclusions

MBTP, known as a combined cognitive and physical training program, improved social communication and white matter integrity among ASD children. The MBTP-related reorganization of brain white microstructures mainly involved regions among the limbic system, midbrain, diencephalon, and cerebrum and covered association (fronto-occipital fasciculus), commissural (corpus callosum and fornix), and projection (corticospinal tracts, fibers in corona radiate, internal capsule, and cerebral peduncle) fibers/tracts. These findings provide further empirical evidence that MBTP is a useful adjuvant therapy to existing standard treatments for patients with ASD. Given that MBTP is a safe, low-cost, and accessible intervention strategy that induces measurable improvements in social communication and neural correlates of ASD, clinicians could consider recommending and integrating MBTP in the treatment of autistic children.

## Figures and Tables

**Table 1:** Mini-basketball training content and goal for each session.

Category	Content	Goal	Duration
Classroom routine	Line up, classroom greetings, roll call, etc.	Communication and social interaction	2 min
Warm-up activities	Stretching, jogging, limbs exercise, etc.	Warm-up	8 min
Mini-basketball training program	Phase I: simple basketball training Phase II: mini-basketball skill learning Phase III: game based on mini-basketball	Social interaction and mini-basketball skills development	25 min
Cool-down activities	Relaxation exercise and summary	Review, summary, reward, clean-up	5 min

**Table 2:** Mini-basketball training program protocol.

Phase	Goal	Content	Duration
Phase I	Standardize classroom routines Increase children’s interest in mini- basketball	Classroom routines (taking turns, waiting, obeying, etc.) Simple basketball training (roll and throw the ball, etc.)	2 weeks
Phase II	Improve children’s mini-basketball skills Improve their social communication skills	Basic basketball skill (dribbling, passing, shooting, etc.) Peer coordination training (passing and catching ball, relay racing, etc.)	8 weeks
Phase III	Improve children’s cooperative ability, social skills, and collectivization	Group game based on mini-basketball (basketball-dribbling relay, basketball-passing relays, basket-moving shooting, playing ducks, etc.)	2 weeks

**Table 3:** Participants’ characteristics (mean ± standard deviation).

Variable	Control Group	Exercise Group
Number	14	15
Gender (boys/girls)	13/1	12/3
Age (years)	4.68 ± 0.72	5.13 ± 0.61
BMI (height/weight^2^)	16.08 ± 1.69	15.65 ± 1.17
CARS ^a^	38.86 ± 3.90	41.20 ± 7.23
CSHQ ^b^	58.00 ± 12.53	56.80 ± 5.06
CEBQ ^c^	53.50 ± 20.49	55.20 ± 7.67

^a^ CARS: Childhood Autism Rating Scale. ^b^ CSHQ: Children’s Sleep Habits Questionnaire. ^c^ CEBQ: Child Eating Behavior Questionnaire.

**Table 4:** Analysis of two groups for social communication variables (mean ± standard deviation).

Variable	Control Group (*n* = 14)		Exercise Group (*n* = 15)		F
	Baseline	Post-Test	Baseline	Post-Test	
SRS-2 T-score ^a^	84.64 ± 20.65	97.14 ± 22.14	96.53 ± 26.28	85.27 ± 29.41	11.869 **
Social awareness	10.36 ± 2.50	11.36 ± 2.68	12.40 ± 3.11	11.87 ± 4.37	3.243
Social cognition	17.21 ± 3.68	19.42 ± 4.01	20.27 ± 5.15	17.07 ± 5.23	11.872 **
Social communication	30.71 ± 8.34	35.43 ± 8.37	33.80 ± 11.37	29.80 ± 10.705	10.094 **
Social motivation	14.36 ± 4.55	14.50 ± 4.35	15.80 ± 4.13	13.47 ± 5.37	2.902
Autistic mannerisms	12.00 ± 5.46	16.43 ± 6.38	14.27 ±6.45	13.07 ± 5.95	6.283 **

Numbers presented are F statistics representing tests of interaction effect of group by time. ^a^ The total score of the Social Responsiveness Scale Second Edition (SRS-2). ** means *p* < 0.01.

**Table 5:** Differences in FA and MD values for specific fiber tracts in an atlas-based ROI analysis between groups (mean ± standard deviation).

WM Region	Control Group (*n* = 14)		Exercise Group (*n* = 15)	
	Baseline	Post-Test	Baseline	Post-Test
Fractional Anisotropy				
Body of corpus callosum	0.557 ± 0.031	0.551 ± 0.038	0.556 ± 0.024	0.497 ± 0.051
Fornix	0.477 ± 0.078	0.463 ± 0.078	0.479 ± 0.055	0.654 ± 0.025
Right cerebral peduncle	0.621 ± 0.024	0.617 ± 0.042	0.637 ± 0.031	0.672 ± 0.025
Left posterior limb of internal capsule	0.656 ± 0.024	0.653 ± 0.034	0.658 ± 0.025	0.672 ± 0.019
Right retrolenticular part of internal capsule	0.557 ± 0.018	0.553 ± 0.023	0.561 ± 0.043	0.582 ± 0.030
Left anterior corona radiate	0.390 ± 0.025	0.388 ± 0.031	0.393 ± 0.028	0.415 ± 0.029
Left superior fronto-occipital fasciculus	0.446 ± 0.029	0.436 ± 0.033	0.450 ± 0.024	0.469 ± 0.041
Mean Diffusivity (10^−3^ **mm^2^/s)**				
Left corticospinal tract	0.799 ± 0.059	0.833 ± 0.012	0.805 ± 0.072	0.761 ± 0.076
Right corticospinal tract	0.793 ± 0.037	0.824 ± 0.010	0.799 ± 0.072	0.744 ± 0.080
Left anterior corona radiate	0.866 ± 0.461	0.871 ± 0.069	0.858 ± 0.047	0.820 ± 0.048
